# Fatores de risco para embolia pulmonar em pacientes com COVID-19 anticoagulados na unidade de terapia intensiva submetidos à angiografia por tomografia computadorizada

**DOI:** 10.5935/0103-507X.20210053

**Published:** 2021

**Authors:** Gonzalo Patricio Briceño-Mayorga, Rocío Gutiérrez, Celine Sotomayor, Matías Ebner, Felipe Allende, Rodrigo Assar

**Affiliations:** 1 Hospital San Juan de Dios - Santiago, Chile.; 2 Faculdade de Medicina, Universidad de Chile - Santiago, Chile.; 3 Faculdade de Ciencias, Universidad Mayor - Santiago, Chile.

**Keywords:** Embolismo pulmonar, Coagulação sanguínea, Infecções, Respiração artificial, Insuficiência respiratória, COVID-19, Meios de contraste/efeitos adversos, Unidades de terapia intensiva, Angiografia por tomografia computadorizada

## Abstract

**Objetivo:**

Avaliar a incidência de embolia pulmonar, seu relacionamento com os níveis de dímero D e outros possíveis fatores associados, além dos efeitos adversos da anticoagulação e meios de contraste.

**Métodos:**

Conduziu-se um estudo de coorte retrospectiva em um hospital público chileno. Foram incluídos os pacientes com idade acima de 18 anos com COVID-19, mecanicamente ventilados na unidade de terapia intensiva, admitidos entre março e junho de 2020. Todos os pacientes receberam tromboprofilaxia com heparina, que foi aumentada até uma dose de anticoagulação com níveis de dímero D acima de 3µg/mL.

**Resultados:**

Foram acompanhados 127 pacientes, dos quais 73 foram submetidos à angiografia por tomografia computadorizada (média de idade de 54 ± 12 anos; 49 homens). Sessenta e dois dos 73 pacientes (84,9%) receberam anticoagulação total antes da angiografia por tomografia computadorizada. Além disso, 18 dos 73 pacientes tiveram embolia pulmonar (24,7%). Na comparação entre pacientes com e sem embolia pulmonar, não se observaram diferenças significantes em termos de idade, sexo, obesidade, tabagismo, escores de Wells e Genebra revisado, dímero D ou mortalidade. O uso de anticoagulantes foi similar em ambos os grupos. O número de dias desde o início da anticoagulação até a angiografia por tomografia computadorizada foi significantemente menor no grupo com embolia pulmonar (p = 0,002). Três pacientes tiveram lesão renal aguda após o contraste (4,1%), e um paciente teve sangramento importante.

**Conclusão:**

Apesar da anticoagulação, um em cada quatro pacientes com COVID-19 submetidos à ventilação mecânica e avaliados com angiografia por tomografia computadorizada apresentou embolia pulmonar. Com uma maior demora para realização da angiografia por tomografia computadorizada após início de anticoagulação empírica, identificou-se um número significantemente menor de embolias

## INTRODUCTION

Infection caused by severe acute respiratory syndrome coronavirus 2 (SARS-CoV-2), also known as coronavirus disease 2019 (COVID-19), presents a wide clinical spectrum from asymptomatic patients or viral pneumonia to more severe conditions, such as severe respiratory distress syndrome, multiorgan failure, and death.^(1)^ Among the biochemical parameters associated with a worse prognosis, coagulation disorders stand out. It has been reported that high D-dimer plasma levels are associated with lower survival in COVID-19 patients,^(2)^ which could indicate a certain predisposition to thrombotic phenomena. Along these lines, a higher incidence of pulmonary embolism (PE) has been reported in critically ill patients with COVID-19 than in patients admitted to the intensive care unit (ICU) for other respiratory causes.^(3,4)^ Given the above situation, the use of anticoagulants in intermediate or high doses has been empirically recommended in high-risk patients.^(5-7)^ A recent retrospective study suggests that anticoagulant treatment could be associated with decreased mortality in patients with severe COVID-19.^(8)^ However, anticoagulation is not free of risks, so its use must be carefully evaluated.^(6)^ The test of choice to diagnose PE is pulmonary CT angiography, a study that is not always feasible to perform in the context of COVID-19 patients, especially those in critical condition.^(6,9)^ In addition, the use of contrast medium carries the risk of certain complications, such as acute kidney injury.^(10,11)^ Knowing the incidence of thrombotic complications in COVID-19 patients and its relationship with coagulation disorders is important to determine the reasoning for anticoagulation use, especially in ICU patients, who are at high risk of thrombosis and bleeding.

Given the aforementioned antecedents, a cohort of COVID-19 patients admitted to the ICU of a tertiary-level center in Santiago de Chile who underwent pulmonary computed tomography (CT) angiography was studied. We aimed to evaluate the incidence of PE and its relationship with D-dimer levels and other possible factors associated with an increased risk of PE, in addition to the presence of adverse effects secondary to anticoagulant treatment and contrast medium.

## METHODS

A retrospective observational study was implemented at *Hospital San Juan de Dios*, a teaching-care center, the oldest in the country, serving a population close to 1.1 million people. Patients who were at least 18 years of age, diagnosed with COVID-19, connected to mechanical ventilation and admitted to the *Hospital San Juan de Dios* ICU between March 24 and June 4, 2020, were included. Diagnostic confirmation of SARS-CoV-2 infection was made by reverse-transcriptase polymerase chain reaction technique in respiratory tract samples. Patients who were extubated before 48 hours, intubated for nonrespiratory causes, were undocumented patients, had CT angiography taken prior to ICU admission, were transferred to another hospital in less than 7 days, or did not have CT angiography performed within their follow-up were excluded. The research was approved by the institution’s Scientific Ethics Committee (Resolution 024667), and given the study’s design, informed consent was waived by the committee.

Patients were followed from their admission to the ICU for 45 days or until their death, transfer to another healthcare center or discharge, so some patients completed their follow-up in the wards. Demographic, clinical, and laboratory data were obtained from electronic medical records and were evaluated in patients with and without PE. In addition, to assess the pretest probability of PE, the Wells score and the revised Geneva score were applied within 24 hours prior to performing CT angiography. For both scales, 3 risk groups were defined. In the case of the Wells score, zero to one, two to six, and > 6 points were defined as low, moderate, and high risk, respectively. For the revised Geneva score, zero to three, four to ten, and >10 points were defined as low, moderate, and high risk, respectively. All patients received thromboprophylaxis during hospitalization, and some of them had been on anticoagulation since their admission due to previous medical conditions. In the case of D-dimer levels higher than 3µg/mL, the heparin dose was increased at anticoagulation doses according to local clinical guides (Table 1).

**Table 1:** Anticoagulation therapy local guide

D-dimer (*µ*g/mL)	Anticoagulant dose
< 3	Dalteparin 5,000UI once a day, subcutaneous*
> 3	Dalteparin 5,000UI, twice a day, subcutaneous (< 80kg) or 7,500UI, twice a day, subcutaneous (> 80kg)†
> 3 and suspicion of PE/DVT, or respiratory deterioration not explained by another cause	Dalteparin 100UI/kg, twice a day, subcutaneous (maximum dose 10,000UI twice a day)†

PE - pulmonary embolism; DVT - deep vein thrombosis.

* If creatinine clearance < 30mL/minute, unfractionated heparin was administered at a prophylactic dose (5,000UI three times a day subcutaneously); † if creatinine clearance < 30mL/minute, unfractionated heparin was administered at a therapeutic dose (target of activated partial thromboplastin time 1.5-2 times control).

### Laboratory tests

The highest D-dimer plasma level (measured using the latex agglutination method) and fibrinogen (using the Clauss method) were registered, and the baseline plasma creatinine value (prior to performing CT angiography) and its highest control value within 48 hours postcontrast were also documented.

### Adverse events

Postcontrast acute kidney injury (PC-AKI) was evaluated as a contrast medium-related adverse effect, defined by a difference between baseline and control plasma creatinine ≥ 0.3mg/dL, according to the Consensus Statements from the American College of Radiology and the National Kidney Foundation 2020.^(12)^ Regarding anticoagulant use, the presence of secondary bleeding was evaluated as an adverse event. The severity of bleeding was defined as determined by the Anticoagulation Control Subcommittee of the Scientific Committee of the International Society of Thrombosis and Haemostasis, determining major bleeding as a fall in hemoglobin ≥ 2g/dL or the need to transfuse ≥ 2 units of red blood cells and/or symptomatic bleeding in a critical organ and/or fatal bleeding. Minor bleeding was considered bleeding that required medical intervention without meeting the criteria for major bleeding.^(13^

### Pulmonary computed tomography angiography

Computed tomography angiography was acquired in both 16- and 64-channel equipment after injection of 70 to 90mL of isosmolar contrast medium using the bolus tracking technique and a trigger threshold between 160 and 250 HU in the pulmonary arterial trunk. Images were reconstructed with a 1mm slice thickness in both pulmonary and mediastinal windows. Radiologists’ reports were reviewed, and the location of PE was classified according to the site of the most proximal luminal defect and if it was unique or multiple. Furthermore, the experience of radiologists was registered as a measure of the number of years since obtaining the specialty to assess whether there was a difference between the positive and negative groups for PE to rule out information bias.

### Statistical analysis

The results are expressed as percentages or means ± standard deviations, as appropriate. The confidence interval (CI) for the PE incidence rate was obtained with the Agresti-Coull methodology. To analyze the influence of each factor on the PE result, Fisher’s exact test (odds ratio - OR values) and the *t*-test for means comparison were used to compare binary and continuous data, respectively.

A p-value < 0.05 was considered statistically significant. Statistical analysis was performed with RStudio software^(14)^ (v 1.2.5033, R v 3.6.3).

## RESULTS

**Figure 1:**
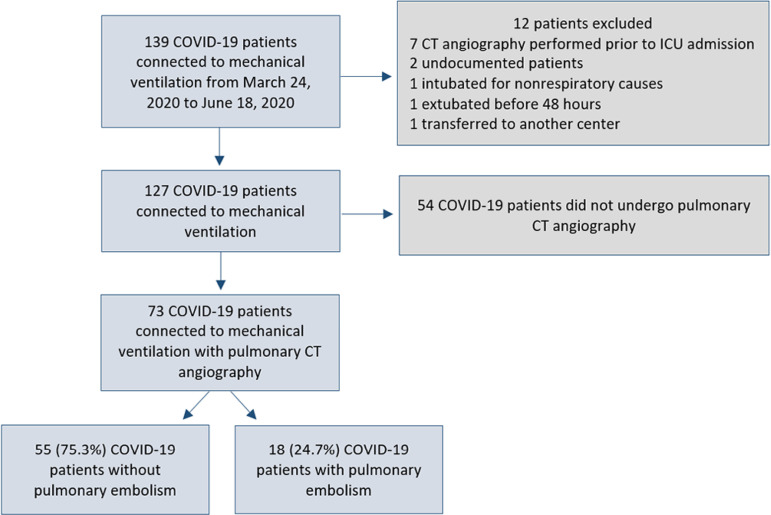
Patient population of the study. CT - computed tomography; ICU - intensive care unit.

During the study period, a total of 139 patients with COVID-19 were admitted to the ICU and were connected to mechanical ventilation. Twelve patients were excluded: 7 with pulmonary CT angiography performed prior to ICU admission, 2 undocumented patients, 1 intubated for nonrespiratory causes, 1 extubated before 48 hours, and 1 transferred to another center within 7 days of admission. Therefore, 127 patients were followed up, of whom 54 did not undergo pulmonary CT angiography within their follow-up period (Figure 1). Finally, 73 patients with CT angiography performed for suspected PE were included (mean age of 54 ± 12 years, 49 men). Prior to CT angiography, 62 of the 73 patients (84.9%) received full anticoagulation doses, and 11 of the 73 patients received prophylactic doses. At the end of the study period, 9 patients had died, reaching a mortality rate of 12.3%, 23 patients remained hospitalized (31.5%), 39 patients had been discharged (53.4%) and 2 patients had been transferred to another hospital (2.7%). It should be noted that all patients underwent CT angiography due to clinical suspicion of PE.

**Figure 2:**
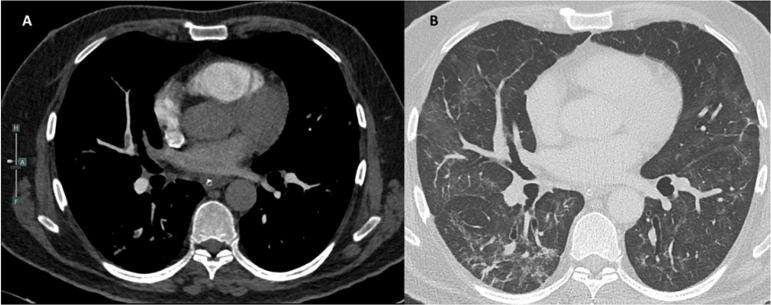
Pulmonary computed tomography of a patient from the studied sample. A filling defect of the segmental branch of the middle lobe pulmonary artery is observed in the mediastinal window (A). Pulmonary window showing features of severe acute respiratory syndrome coronavirus 2 pneumonia (B).

A total of 18 cases of PE were detected with an incidence of 24.7% (95%CI 16.1 - 35.7%), of which 10 had multiple locations (56.6%). Regarding the affected arteries, 11 were segmental (61.1%), 4 were subsegmental (22.2%), and 3 were lobar (16.7%). One case of segmental PE is shown in figure 2. When comparing patients with and without PE, no differences were observed in age, sex, or mortality. Two patients with PE (2/18, 11.1%) and 9 without PE (9/55, 16.4%) died. Patients with PE showed a trend toward a lower frequency of obesity (50% *versus* 61.8%). Although not statistically significant, sample size, smoking, and the Wells and revised Geneva scores were the most predictive factors of PE. A higher frequency of smoking (33.3% *versus* 21.8%, OR of 1.75), a moderate or high Wells score (100% *versus* 89.1%, OR of 1.3), and a moderate or high revised Geneva score (83.3% *versus* 76.3%, OR of 1.33) were obtained for patients with PE *versus* without PE. The use of full anticoagulation was similar in both groups (83.3% *versus* 85.5%).

Regarding laboratory data, there was a trend toward higher D-dimer levels in patients with PE (mean 8.6µg/mL ± 4.8 *versus* 7.9µg/mL ± 5.7), without being significant. Peak fibrinogen levels were significantly lower in patients with PE (mean 739.8mg/dL ± 124.6 *versus* 829.8mg/dL ± 214.6; p = 0.02). The clinical and laboratory characteristics of the patients are summarized in table 2.

**Table 2:** Clinical, demographic, and laboratory characteristics of the patients

Characteristic	Total (n = 73)	Pulmonary embolism present (n = 18)	Pulmonary embolism absent (n = 55)	p value
Age	54.3 ± 12.5	51.2 ± 13	55.3 ± 12.3	0.12
Sex				0.59
Male	49 (67,1)	12 (66,6)	37 (67,3)	
Female	24 (32.9)	6 (33.3)	18 (32.7)	
Body mass index				
< 30	30 (41.1)	9 (50)	21 (38.2)	0.27
≥ 30	43 (59.9)	9 (50)	34 (62.8)	
Smoking actively	18 (24.7)	6 (33.3)	12 (21.8)	0.26
Wells score for PE*				
Low	4 (5.5)	0 (0)	4 (7.3)	0.30
Moderate or high†	67 (91.8)	18 (100)	49 (89.1)	
Geneva revised score*				
Low	14 (19.2)	3 (16.7)	11 (20)	0.49
Moderate or high†	57 (78.1)	15 (83.3)	42 (76.3)	
Anticoagulation prior to CT angiography				
Therapeutic dosing	62 (84.9)	15 (83.3)	47 (85.5)	0.55
Prophylaxis dosing	11 (15.1)	3 (16.7)	8 (14.5)	
Highest D-dimer value (0.5*µ*g/mL)	8 ± 5.5	8.6 ± 5.6	7.9 ± 5.7	0.23
Highest fibrinogen value* (mg/dL)	808.9 ± 200.2	739.8 ± 124.6	829.8 ± 214.6	0.02

PE - pulmonary embolism; CT - computed tomography.

* There were missing values for Wells score (2), revised Geneva score (2), and highest fibrinogen value (4); † for both risk scales, the moderate- and high-risk groups were grouped into one group in search of an association with pulmonary embolism. The normal D-dimer level was less than 0.5*µ*g/mL, and the normal fibrinogen level was between 150 and 350mg/dL. Results expressed as median ± standard deviation or number (%).

In relation to time from the highest D-dimer plasma levels to CT angiography, no significant differences were observed between the two groups. In contrast, when evaluating the number of days from ICU admission to CT angiography (17.9 ± 9.7 *versus* 23±9.9; p = 0.03) and the days from the start of anticoagulation until CT angiography (7.5 ± 8.2 *versus* 14.8 ± 10.9; p = 0.002), they were significantly lower in the PE group. The experience of the radiologists who reported the images was 5.1 ± 2.8 and 5 ± 3.7 years in patients with and without PE, respectively, with no significant differences. The data are summarized in table 3.

**Table 3:** Characteristics of pulmonary computed tomography angiography and anticoagulant therapy

Characteristic	Total (n = 73)	Pulmonary embolism present (n = 18)	Pulmonary embolism absent (n = 55)	p value
Days from highest D-dimer until CT angiography	15.2 ± 5.9	14.5 ± 5	15.4 ± 6.2	0,09
Days of anticoagulation treatment prior to CT angiography	13 ± 10.7	7.5 ± 8.2	14.8 ± 10.9	0,002
Days from ICU admission until CT angiography	21.8 ± 10	17.9 ± 9.7	23 ± 9.9	0,03
Thrombus location				
Lobar		3 (16.7)		
Segmental		11 (61.1)		
Subsegmental		4 (22.2)		

CT - computed tomography; ICU - intensive care unit. Results expressed as median ± standard deviation or number (%).

Finally, concerning adverse events, three patients presented PC-AKI (4.1%). Only one patient presented bleeding in relation to anticoagulation use and suffered a chest wall hematoma with criteria for major bleeding. None of the patients with adverse events had PE.

## DISCUSSION

Our study showed that 24.6% of pulmonary CT angiography performed in patients with COVID-19 hospitalized in the ICU presented PE. This incidence is consistent with previous studies in COVID-19 patients undergoing CT angiography (22% - 37%).^(15-19)^ However, these studies considered both ward and ICU patients. When analyzing only ICU patients, the incidence of PE ranges from 25 to 50% in patients undergoing CT angiography,^(3,18)^ despite receiving prophylactic anticoagulation. The incidence of our study is at the lower limit of the range, which could be explained by the long anticoagulation time before CT angiography and the prolonged period between ICU admission until CT was performed.

The high incidence of PE reported in our study was detected even though 84.9% of patients were anticoagulated, which reflects the high risk of thrombotic complications in patients with COVID-19. Therefore, some authors propose anticoagulation use in high-risk critically ill patients,^(5-7)^ considering that reported bleeding complications associated with its use are infrequent,^(8,20)^ as in our study where only one patient had major bleeding. In addition, the most significant finding was that with a longer delay in performing CT angiography, once empirical anticoagulation was started, significantly less PE was identified. This fact could reflect a sub diagnosis considering that studies are carried out in patients under treatment. This has an impact on therapeutic actions since anticoagulation would be suspended in patients without PE findings, which in some cases could lead to undertreatment.

Contrary to previous reports,^(15-18)^ we did not find an association between D-dimer levels and PE. This could be explained since these studies included patients hospitalized in wards, in addition to patients in the ICU, highlighting that in the groups with PE, there was a higher percentage of patients in the ICU compared to the groups without PE, unlike our cohort which included only ICU patients. Further potential explanations are type II error due to limited sample size, and the confounding effect of full-dose anticoagulation started days before CT angiography.

It has been reported that ICU patients have higher D-dimer levels than non-ICU patients;^(21)^ moreover, high levels of D-dimer are associated with a greater degree of inflammation in patients with COVID-19,^(22)^ and therefore, patients in more serious conditions would have higher D-dimer levels. Consequently, D-dimer levels would have a limited role in predicting thrombosis in critically ill patients, given its low specificity.

With respect to fibrinogen levels, elevated values have been reported in patients with COVID-19 in the context of acute inflammation and are described as endothelial dysfunction and a prothrombotic factor.^(23,24)^ Regardless of what has been described in the literature, our cohort showed that patients with PE had significantly lower peak levels of fibrinogen than patients without PE. This association has not been previously reported, and we found no explanation for this phenomenon. Future studies are needed to clarify this association.

Referring to the safety of CT angiography, in our cohort, it was a safe diagnostic method, given the low number of patients who had PC-AKI. However, it is important to consider the risk for healthcare workers during the transport of COVID-19 patients to receive CT, as well as the risk of decompensation and instability for the patient, derived from the transfer of a critically ill patient.^(25,26)^

Regarding the scores to evaluate pretest probability, neither the Wells score nor the revised Geneva score was helpful in predicting PE in the ICU. Another study carried out in COVID-19 patients also found no association between a likely Wells score value and the presence of PE,^(17)^ which would indicate that application of these predictive scores would have limited utility to distinguish which patients should undergo imaging studies to confirm PE. Although the OR values for each PE risk factor were obtained separately, with this analysis, we took the first step toward a multiple logistic predictive model, which will make sense with the fit when we reach a larger sample size.

Our study is a retrospective observational study and has several limitations. As the evaluation of thromboembolic complications was not standardized, there was a long period until CT angiography was performed, so most patients remained anticoagulated for the entire waiting time, which could affect the incidence of PE and was probably underestimated. Conversely, an additional limitation of this study is that not all patients underwent CT angiography, only those with a clinical suspicion of PE. This characteristic of the study would likely overestimate the incidence of PE. Additionally, there was no protocol for collecting laboratory data, which could have affected the investigation of peak plasma levels of D-dimer and fibrinogen. Therefore, to evaluate PE incidence and its relationship with D-dimer levels, prospective studies that include a greater number of patients are needed and should be carried out.

## CONCLUSION

Despite anticoagulation, one in four COVID-19 patients connected to mechanical ventilation and evaluated with pulmonary computed tomography angiography had pulmonary embolism. The days from intensive care unit admission and the start of anticoagulation to computed tomography angiography were significantly lower in the group with pulmonary embolism. Nevertheless, we did not find a significant statistical relationship with the recommended predictive scores for pulmonary embolism or with D-dimer levels. Further studies with a larger number of patients are necessary to accept or refute these findings.
